# The Evolutionary Game of Post-conflict Management for New Generation of Construction Workers in China: The Mediating Role of Foremen

**DOI:** 10.3389/fpsyg.2022.950387

**Published:** 2022-07-11

**Authors:** Junlong Peng, Qi Zhang

**Affiliations:** School of Traffic and Transportation Engineering, Changsha University of Science and Technology, Changsha, China

**Keywords:** post-conflict processing, evolutionary game theory, affective event theory, foreman influence, emotional perception

## Abstract

The emotional perception of the new generation of Chinese construction workers is becoming stronger, and the traditional punishment-type management model is gradually failing. In order to address the safety hazards caused by the negative emotions generated after workers' conflict events, the motivation of workers to actively participate in the construction of safety climate is increased, and the safety performance of construction projects is enhanced. This paper introduces emotional event theory to assess workers' psychological perceptions and uses foreman as an intermediary for safety management to analyze the decision-making process between managers and work-groups in the safety management process. By establishing a tripartite evolutionary game model of manager, foreman, and worker, the evolutionary differences among the three parties when the manager is strict or appeasing are examined. The results of the study showed that managers who showed appeasement were more effective than those who showed stringency in accomplishing the safety goals of the project. As the workers' psychological perception index increased, workers were more inclined to adopt aggressive strategies, and their behavior was more influenced by their own moral identity as well as the foreman's attitude under the manager's appeasement attitude. This study can provide managers with suggestions on how to handle the situation after a conflict, which can help regulate the behavior of construction teams and eliminate safety risks.

## 1. Introduction

The construction industry has steadily become a high-risk industry due to its complicated operational environment, frequent usage of harmful appliances, and the inherent decentralization and mobility of construction employees (Fang et al., 2006). In China, for example, there were 6,005 catastrophic accidents and 7,275 deaths in the construction industry between 2010 and 2019 (Xu and Xu, 2021). The 2018 report on safety in China (Ministry of Emergency Management of the People's Republic of China, 2018) pointed out the poor supervision of safety hazards by participating parties and the prevalence of construction site violations, which directly lead to accidents. Many of these safety hazards are caused by conflicts and disputes (Harmon, 2003), which can lead to project delays and even casualties if conflicts are not handled properly (Jaffar et al., 2011). Casualties are the most unacceptable tragedy in the construction process, and the repeated safety accidents reflect the failure of safety management and the deficiencies in safety performance. Due to this, safety management has been put at the top of many construction projects. Many research has been undertaken to investigate the important factors that contribute to construction safety risks, and these studies point to the interaction between project managers and construction employees as a key determinant (Shao et al., 2019; Zhou et al., 2019; Li et al., 2021).

As China's modernization process accelerates, the number of building projects grows, and a shortage of construction workers prompts an increasing number of migrant workers to consider the construction industry as a way to make a living (Swider, 2015). In construction projects, however, maintaining a balance between the management team and the construction teams is difficult, the number of workers outnumbers the number of managers, and the managers are unable to maintain constant command and direction, of overall workers. The huge groups of employees are also divided into many smaller teams based on the type of work due to the decentralized and mobility character of construction workers, as well as the technical requirements of construction. The advancement of the construction process necessitates the cooperation of each team, and the restricted construction site and construction supplies can easily lead to conflicts between the construction teams, increasing the risk of injury (Forteza et al., 2022). Especially in work at height, conflict incidents create a discordant safety climate and keep the safety bottom line at an extremely low level (Wong et al., 2016). The concept of safety climate has its roots in organizational culture (Zohar, 1980) and is often used to describe the perceptions and attitudes of employees toward safety issues (Guldenmund, 2000). Because the team is working temporarily and is unfamiliar with each other, post-conflict antagonism is high, the perception of cooperation is low, and cooperative behavior is negative (Liu et al., 2021). Workers' own will may override their safety consciousness under the impact of such emotions, causing them to act in ways that may result in safety incidents (Liu et al., 2020).

Maintaining worker safety awareness as a top priority is a difficult task for safety managers, and a great management strategy is essential. However, research on this problem is far from thorough. Although many studies have been conducted to reduce workers' unsafe behaviors (Smith et al., 2017; Ma et al., 2021), there are fewer dynamic analyses from the perspective of a construction team, in which managers divide all workers into teams and adjust management measures dynamically based on the characteristics of each team and the state of construction safety awareness. Some research findings have proposed influencing construction workers' behavior from a management level perspective (Zhu et al., 2021; Zulu and Khosrowshahi, 2021), these studies only treat workers as a whole, and the constant turnover of shifts in short- and medium-term construction projects makes it difficult to transfer management's organizational characteristics to the construction team, and there is a great deal of uncertainty about whether workers' personalities fit together.

Although accidents in the construction industry are not uncommon, but narrowing the perspective to specific construction projects, the casualty rate of accidents is still a small number compared to the number of employees. Under this premise, workers will subjectively ignore safety risks and take some more convenient but dangerous behaviors. Although the safety manager has overall control over the safety performance of the project, he cannot always pay attention to the construction personnel in the details. Their bounded rationality and incomplete grasp of background information make evolutionary game theory useful in this context. As a result, in order to complement research on the causes and treatments of workers' unsafe behaviors, this study hypothesizes that the factors affecting project safety performance and effective treatment measures will be found under the two attitudes (Strict supervision and Appeasing supervision) of managers following the team conflict event, as well as the constraints and strategies to promote the evolutionary stability of the game, which is the excellent safety performance expected by the stakeholders. The main contributions of this paper are as follows: (1) introducing workers' psychological identity as a tendency indicator for judging workers' unsafe behaviors, and using evolutionary game theory to analyze the mutual influence of safety managers, foremen and workers. (2) Exploring the effectiveness of safety managers' strict supervision and appeasing supervision in treating the new generation of construction workers. (3) To reveal the intermediary role of foreman between safety managers and workers in safety management.

The following are the remaining sections of this paper: The second section of the paper is devoted to a review of the literature. Section 3 examines the game equilibrium and constructs an evolutionary game model based on replicated dynamic systems. The simulation study for various scenarios is presented in Section 4. Then, Section 5 discusses the simulation results and the proposed measures for a safe environment. Finally, in Section 6 we provide our conclusion of the work.

## 2. Literature Review

Construction-related safety incidents are not unavoidable, and significant progress has been accomplished in recent decades. Interviews, surveys, data mining, and game theory models have all been used to study the parties engaged in construction.

### 2.1. Unsafe Behavior and Affective Events Theory

At present, many researchers have indicated that workers' unsafe behaviors might have a considerable detrimental impact on safety performance (Mitropoulos et al., 2005, 2009; Choudhry, 2014). And majority of academics are interested in learning more about the reasons for insecure behavior (Wu et al., 2018; Fang et al., 2020). Mazzetti et al. (2020) proposed that construction workers' safety behavior is related to their safety perceptions and knowledge. Yao et al. (2021) reached similar conclusions after analyzing 6,561 tweets about construction safety on Twitter, and in addition, they suggested that the government as an opinion leader can act as a medium for safety knowledge dissemination and raise construction workers safety awareness. These research results analyze the results of external influence on the behavior of construction workers and the ways in which they are affected, but do not pay much attention to the beginning and process of workers' change.

Therefore, some scholars have begun to build simulation models to evaluate the entire process of building accidents in order to better extract the variables that cause accidents and investigate the handling procedures that can help to maintain good safety performance. For construction accidents, Ge et al. (2022) looked at the application of five common accident causation models in China from 1978 to 2018, finding the significant influence of previous modeling research on policy formation, accident investigation and treatment, and safety management measures in the Chinese government. Cabello et al. (2021) further analyzed the various phases of engineering construction to point out the priority of outsourcing variables in accidents and demonstrated the importance of risk assessment.

However, Construction teams are collected temporarily and only work for a limited time in the project due to the mobility and decentralization of construction workers, and there is no close interaction between crews (Fang et al., 2006). Also, construction projects are mostly carried out in resource-limited situations (Du et al., 2021). Conflict events can readily develop between construction teams to vie for resources or seize work locations, and conflict events can cause emotional reactions in individuals, which can further impact their attitudes and behaviors (Weiss and Cropanzano, 1996), increasing the likelihood of safety mishaps (Man et al., 2021).

The core concept of affective event theory is that the events an individual experiences at work affect his or her emotional state, which in turn affects his or her attitudes and behaviors (Weiss and Cropanzano, 1996). Negative events in the workplace are important emotional events that affect employees' emotional state (Bono et al., 2013). Conflict events in construction teams can be categorized as workplace deviant behavior, which is defined as intentional behavior by employees that is detrimental to the organization and other members. This behavior is a specific manifestation of the affective event theory. The affective event theory states that after a dispute arises, the employee's level of moral identification influences his or her emotions and, as a result, his or her subsequent action (Schaumberg and Flynn, 2021). Chen et al. (2020) investigated the attitudes and behaviors of 143 construction workers in the face of family conflict and work environment disruptions, stressing the mediation impact of emotional feelings on work. Employees give greater attention to their own feelings and prefer to make decisions based on their own perceptions, according to Ming and Yue (2018), and the prevalence of deviant behaviors in the workplace is dependent on employees' own perceptions. Kong and Kim (2017) proposed that employees who possess a high degree of psychological mastery have some innovative responses to workplace deviant behavior.

### 2.2. Impact of Foreman

For construction projects, the construction team is the basic unit of operation. During the construction process, the foreman is the main person who directs the workers as well as communicates. Thus, the construction team leader's leadership abilities will have a direct impact on the team's climate. The team leader's safety knowledge will affect the workers' construction conduct and play an essential role in preventing safety mishaps (Hinze, 1981).

With the accelerated rate of urbanization in China, more and more young migrant workers are entering the construction industry (Chen et al., 2018). Younger workers are less safety-conscious than older workers, have a higher risk of being exposed to dangers, and have a negative opinion of the safety atmosphere (Meng and Chan, 2020). These groups have distinct group characteristics, such as a greater sense of fairness and protection of rights (Franceschini et al., 2016), as well as stronger personalities (Tang et al., 2020). Strong punitive measures (fines or other measures) may not be effective in inducing a change in their consciousness, and the role of opinion leaders is more likely to be flanked by them (Choi and Lee, 2018).

Many scholars believe that accidents occur not only as a consequence of employees' unsafe behavior but also as a result of the team leader's negative attitude toward unsafe behavior, which causes team members to pay less attention to their behavior, resulting in tragedies, whereas the team leader's positive influence will ensure a high level of safety. Wang et al. (2021) proposed that workers' safety responsibilities and safety trust in their supervisors mediated the relationship between employees' beliefs about the mutual fulfillment of their safety obligations with their supervisors and their active participation in safety construction. Xiong et al. (2018) studied the perceptions of 586 scaffold workers about employee influence, applying the idea of opinion leaders to construction safety and proving the powerful influence of team leaders on the typical worker. Kaskutas et al. (2016) evaluated safety performance before and after team leader training and discovered that safety scores improved in all aspects after training, demonstrating that effective education of employees by team leaders plays an important role in reducing safety accidents.

The tendency of workers to follow opinion leaders is noted, but how to use this characteristic effectively becomes a challenge. Workers' strong personalities can counter the strict management of safety managers. As a result, many scholars have begun to study the impact of shifts in safety managers' attitudes toward workers on project safety performance. He et al. (2021a) suggests that excellent leadership and communication skills of safety personnel can create a supportive safety climate at the worksite to improve construction safety performance. He et al. (2021b) proposed that worker-safety manager communication is a key variable in safety performance and illustrates the need for enhanced psychological interventions for workers. Liu et al. (2020) pointed out that when leader-member exchange has high quality, it can effectively inhibit the generation of workplace deviant behaviors. The utility of communication in managing safety among the new generation of construction workers is becoming apparent, and several studies have shown that improvements in safety performance can be achieved through foreman-worker interactions. Kaskutas et al. (2013) evaluated the effect of foreman training in safety communication on improving worker safety performance. Kines et al. (2010) suggested that increased foreman-worker verbal safety communication has a significant and long-lasting effect on safety performance improvement. The weight of enhanced communication management by safety managers through the intermediary role of foremen is called appeasing supervision in this paper.

In summary, the above studies demonstrate the influence of emotional perception on worker behavior and the strong influence of foremen on workers. However, the reduced effectiveness of traditional punitive measures (such as fines, reprimand, etc.) still gives construction companies a sense of crisis, and changes in safety management measures are necessary as factors that can directly affect whether a project is carried out or not.

## 3. Research Methodology

### 3.1. Description of Research Framework

Observed through the lens of affective event theory, the nature of conflict behavior that occurs between construction crews at the construction site is an extension of the workplace deviant behavior of workers. The close-knit nature of construction crews makes workplace deviant behaviors less about single person-to-single person influence and more about group-based confrontation. During this period, workers are simultaneously influenced by their own emotional perceptions as well as environmental drivers, which are susceptible to adverse effects on the unavoidable synergy in construction.

Nowadays, with highly popular education in China, the new generation of construction workers is becoming more individualized and more sensitive to emotional perceptions. Whether, they are the perpetrators or recipients of workplace deviant behavior, they are often influenced by the intrinsic mechanism of their own emotions to take positive compensatory measures or negative avoidance attitudes in their subsequent work. Positive remedial measures enhance the safety climate, while negative attitudes produce consequences such as reduced motivation, slower work speed, and the development of unsafe behaviors.

For safety managers, they are unable to reach the emotional perception of all workers. Even when they perceive workers' emotions, they let them go out of the traditional handling mindset. The external safety equipment and measures at construction sites are so complete that the mental health of employees has reached a point where it cannot be ignored as a percentage of construction safety. However, safety managers are reluctant to pay in this area and ignore the mental health of their employees.

Foremen are between managers and workers. On the one hand, they are the leaders of workers, and they live with them day and night, and their emotions and behaviors greatly influence workers; on the other hand, they are still workers in the eyes of managers, and their management style has not changed much. It is obviously unreasonable for Foremen, as intermediary figures between workers and managers, to be treated the same as workers.

These three parties have intersecting interests in safety performance and influence each other. For example, workers' unsafe behaviors that create safety hazards increase the supervision of managers. When both the foremen and workers work negatively, safety accidents are highly likely to occur. In addition, the strategies of foremen, workers, and safety managers are not set in stone in dealing with workgroup conflict. Effective management mechanisms and control of the psychological state are the keys to an ideal safety strategy. Maintaining the awareness of workers' safety first, the positive attitude of the foremen infecting the team, and the effective motivation of the manager to the team members.

Evolutionary game theory assumes that humans are not fully rational objects and at the same time do not possess complete background information. In the game equilibrium, the system reflects a dynamic equilibrium. The setting of various parameters and changes in their values affect the strategy choice of each player. In addition, unlike classical game theory, the participants reach the game equilibrium by trial and error during the evolutionary process. Therefore, evolutionary game theory can be a good way to analyze the relationship between the three players. In this paper, we use evolutionary game theory to determine the dynamic equilibrium of safety managers, foremen and workers to find stable constraints and the relative magnitude of influencing factors to achieve excellent project safety performance.

**Figure 1:**
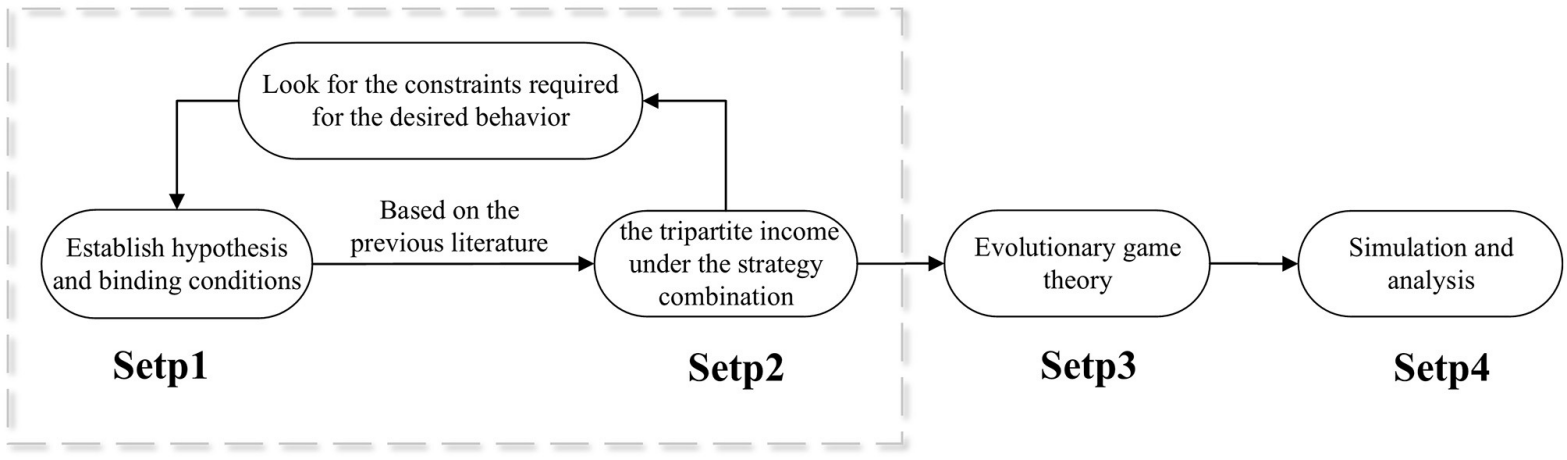
Model frameworks.

The model framework of this paper is shown as follows: (1) Establish the hypothesis to quantify the strategic gains and losses of the three parties. (2) List the difference between the gain and loss of the three parties under the traditional attitude and the appeasement attitude of managers. (3) Calculate the evolutionary stability of each strategy combination and derive the stability conditions. (4) Conduct simulation to verify the stability of the strategy combinations. The entire model framework is shown in Figure 1.

### 3.2. Model Assumptions

Although conflicting behavior is temporarily harmless, managers and members of workgroups will sense impending danger. When confronted with uncertainty and risk, managers and workgroups display limited rationality and emotional drive, causing them to alternate between “self-interest” and “active participation” resulting in various engagement tactics. In addition, this section defines the game player and strategy in positive and negative terms, proposes parameters that affect the utility of the game player, and constructs the payoff matrix of the post-conflict contextual game based on the literature in Table 1 and the literature review in Section 2.

**Table 1:** Review the game and influence of participants on safety performance.

**Literature**	**Players**	**Strategies**	**Impact on safety performance**
Gao et al. (2020)	Government Enterprise	Active regulation/ compliance	In the case of information asymmetry, companies lack trust in regulatory policies and have a low willingness to comply with safety regulations.
Guo et al. (2021)	Government Enterprise Worker	Encourage/provide/ participate in safety training	It demonstrates the importance of government oversight for companies and workers to participate in safety skills training.
Yun et al. (2021)	Tower crane users maintenance parties supervisors	Regulatory compliance/ strict maintenance/ active supervision	The three parties will select a safety regulatory strategy that is favorable to the tower crane operation when the sum of the penalty amount and safety incentive performance is greater than the safety input cost.
You et al. (2020)	Mine owners safety regulators general miners	Strict inspection/ strict supervision/ compliance with rules	Increasing the intensity of rewards and punishments can quickly reduce the rate of unsafe worker behavior.
Gong et al. (2021)	Mine owners safety regulators general miners	Dynamic/ static supervision	The effectiveness of the government's dynamic regulatory mechanism to improve the efficiency of supervision and improve the initiative of enterprises to participate in the construction of safety.

The following assumption are proposed based on the relationship between managers, foremen, workers, the theory of emotional events, and the features of the team's role.

**Assumption 1:** There are two strategic options available to each of the three parties in the game. The probability that the manager will utilize the two strategies of {Strict supervision, Appeasing supervision} is *x* and 1 − *x*, respectively. “Strict supervision” refers to the use of strong punitive measures (e.g., fines), while “Appeasing supervision” refers to giving the foreman psychological pressure through safety education and other means to influence the workgroup members. The foreman's strategy set is {Tough, Lenient}, denoted as the attitude toward the workers with probability *y* and 1 − *y*. Workers are direct actors in team conflict, and they have two alternatives for dealing with the post-event safety climate: {Active participation, Passive response}, with a probability of *z* and 1 − *z*.

**Assumption 2:** Workers acquire guilt after encountering team conflict, according to Schaumberg and Flynn (2021), which impacts their own negative emotions, which in turn influences their job conduct. The moral identity of the workers can have a significant impact on the psychological to behavioral shift. In this game, indicators of moral identity influence the rewards and penalties that workers get. This is expressed in this paper as dissatisfaction *A*_1_ with the tough punishment of the safety supervisor and psychological compensation *A*_2_ for the lenient treatment of the foreman.

**Assumption 3:** The new generation of construction workers, according to Xiong et al. (2018) and Ni et al. (2020), has a strong sense of individuality and is resistant to harsh punishments; the foreman, as the spiritual leader of the workers, has more control over this group of workers, and the foreman's attitude influences the workers' psychology and behavior. In other words, workers are influenced by the attitudes of both managers and foremen.

**Assumption 4:** The workers' attitude toward the construction of safety climate affects the probability of safety accidents. The probability of an accident with a positive attitude is α_1_, and the probability of an accident with a negative attitude is α_2_ (α_2_ > α_1_). The accident will cause huge losses to all three parties. The government will impose a fine of *P*_1_ on the safety supervisor; the construction company will deduct the bonus of the foremen *P*_2_; and the workers will suffer a safety loss of *P*_3_. Under the strict supervision of the safety managers, the two strategies of the foreman represent fines to be borne by himself or by the workers, triggering either praise or dissatisfaction from the workers, expressed as reputation gain or loss *R*. Under the appeasing management of the safety managers, the forgiving attitude of the foreman needs to bear the pressure from the safety managers *K*, the cost of tough management is *C*_2_. The two strategies of the safety managers cost *C*_11_ and *C*_12_, respectively, to manage, and in a good safety climate will receive a bonus *I*.

With the above assumptions, the parameters and variables of this tripartite evolutionary game model are shown in Table 2.

**Table 2:** Explain of the parameters.

**Parameters**	**Explain**
*C*_11_	Management costs for safety managers choosing a Strict supervision strategy.
*C*_12_	Management costs for safety managers choosing a Appeasing supervision strategy.
*I*	Bonuses for safety managers due to good safety climate.
*F*	Fines issued by the safety manager to the working group.
*R*	The foreman's reputation is lost or gained.
*K*	The psychological pressure gained by the foreman choosing the Lenient strategy.
*C*_2_	The management costs by the foreman in choosing the Tough strategy.
*P*_1_	Penalties for safety managers after a safety incident.
*P*_2_	Penalties for foreman after a safety incident.
*P*_3_	The safety loss of workers after a safety accident.
*T*	The additional cost to workers of choosing an Active participation strategy.
α_1_	The probability of a safety incident when workers choose the Active participation strategy.
α_2_	The probability of a safety incident when workers choose the Passive response strategy.
*A*_1_	Workers' dissatisfaction with safety manager punishment is influenced by their own moral identity.
*A*_2_	Workers' approval of the foreman's behavior is influenced by their own moral identity.

### 3.3. Model Establishment

The traditional way of safety management is for managers to inspect the construction process and results of workers from time to time. In the process, the manager finds the unsafe behavior of workers or other violations of regulations and takes strong measures such as simple fines. However, managers are lagging behind in dealing with the situation according to the site, and safety hazards bring the possibility of safety accidents once they arise. And tough punishments are difficult to achieve satisfactory results in the face of employees with strong personalities. If managers change the way of handling and do not handle the workers hard and directly, the foremen should play an intermediary role between managers and workers. When the safety manager chooses the Strict supervision strategy, the construction team has to bear the fines from the safety management, and the foreman's attitude determines who pays the fines, and the foreman's and the workers' fear of fines affects their strategy choices. When the safety manager chooses the Appeasing supervision strategy, the substantial fines are transformed into psychological pressure on the foreman, and in this case, the workers' strategy choice is mainly determined by their own moral identity influenced by the psychological compensation to the foreman as well as the safety manager. Enhance safety education and investment to the foreman, let the foreman influence the safety status of workers, and assist the manager to coordinate and manage after the conflict occurs. The foreman is the manager of the workers and a participant in the work, and they can be the first to know the condition of the construction site. Managers can quickly learn from foremen about real-time safe production, quickly calm workers' emotions and adjust management measures (Kaskutas et al., 2016). In this case, the regulatory results are more flexible and effective for the new generation of self-reliant construction workers.

**Figure 2:**
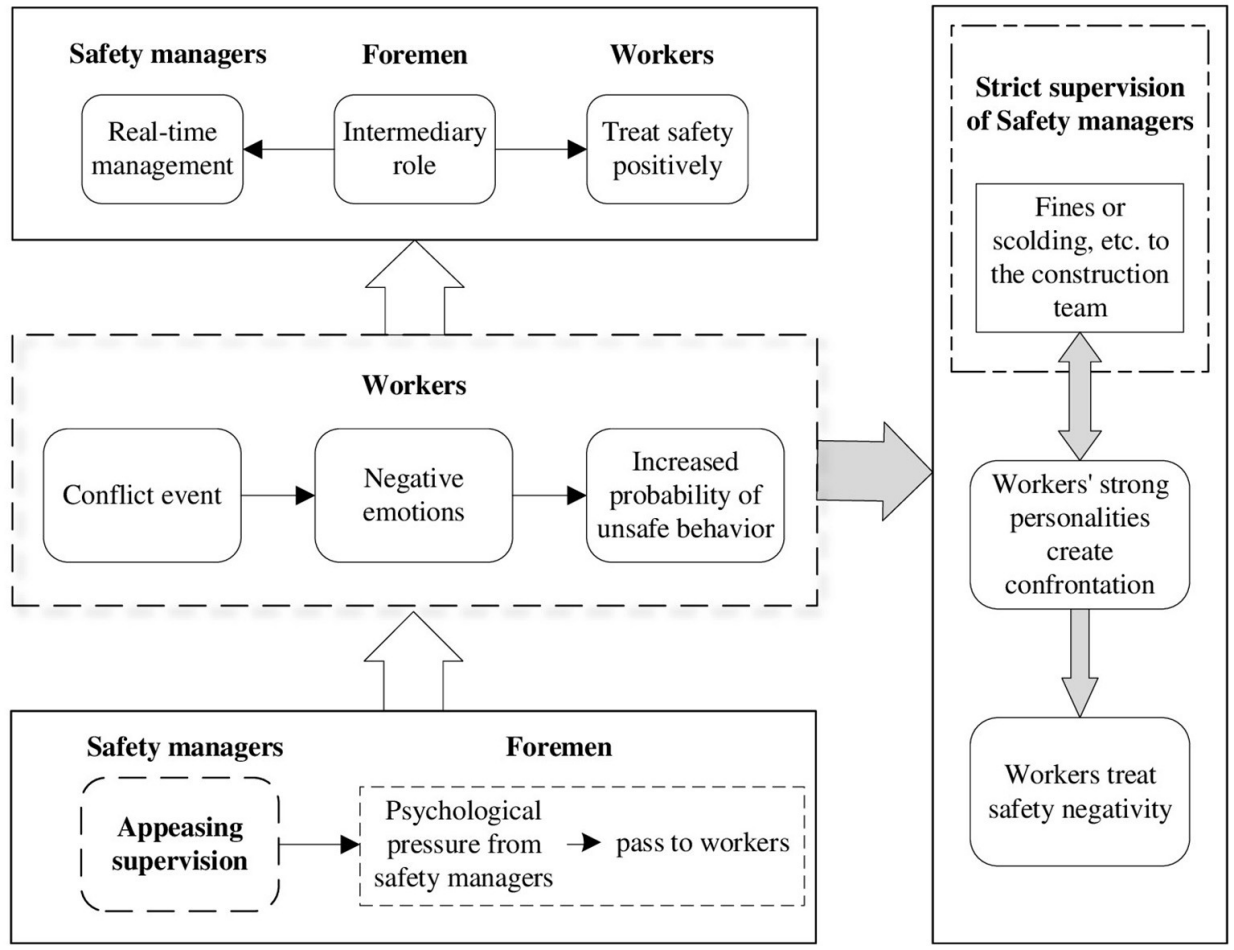
Tripartite relationship diagram.

According to the above discussion, combined with literature mining and the actual situation, the tripartite relationship diagram is obtained, as shown in Figure 2.

Evolutionary game theory is carried out on the premise that the three parties belong to limited rationality. The strategy choices of the three parties are influencing each other and choosing the most favorable strategy in perception. Both foremen and workers have two strategies to deal with the strict supervision or appeasing supervision of safety managers, which will produce a combination of eight strategies. Based on the above assumptions, the profits and losses of the three parties are calculated under eight strategy combinations.

(1) When the safety manager selects Strict supervision, the foreman selects Tough and the worker selects Active participation. Safety managers need to pay management costs and possible safety rewards or penalties, and their benefits are −*C*_11_ + (1 − α_1_)*I* − α_1_*P*_1_. If the foreman chooses to let the workers accept punishment, he will suffer loss of reputation, and the income is −*R* − α_1_*P*_2_. Workers' active participation in safety construction requires additional construction costs, and the income is −*T* − *F* − α_1_*P*_3_.

(2) When the safety manager selects Strict supervision, the foreman selects Tough and the worker selects Passive response. At this time, the safety risk will increase to α_2_. Safety managers need to pay management costs and possible safety rewards or penalties, and their benefits are −*C*_11_ + (1 − α_2_)*I* − α_2_*P*_1_. If the foreman chooses to let the workers accept punishment, he will suffer loss of reputation, and his income is −*R* − α_2_*P*_2_. Workers' dissatisfaction with the tough punishment of safety managers will produce *A*_1_ psychological compensation under the workers' Passive response Strategy, and its benefit is −α_2_*P*_3_ + *A*_1_ − *F*.

(3) When the safety manager selects Strict supervision, the foreman selects Lenient and the worker selects Active participation. Safety managers need to pay management costs and possible safety rewards or penalties, and the benefits are −*C*_11_ + (1 − α_1_)*I* − α_1_*P*_1_. The foreman took the fine and received the reputation gain from the workers, which improved his prestige in the workegroup, with a gain of *R* − *F* − α_1_*P*_2_. The active participation of workers will produce psychological compensation *A*_2_ for the foreman, and the benefit is −*T* + *A*_2_ − α_1_*P*_3_.

(4) When the safety manager selects Strict supervision, the foreman selects Lenient and the worker selects Passive response. At this time, the safety risk will increase to α_2_. Safety managers need to pay management costs and possible safety rewards or penalties, and their benefits are −*C*_11_ + (1 − α_2_)*I* − α_2_*P*_1_. The foreman took the fine and received the reputation gain from the workers, which improved his prestige in the workgroup, with a gain of *R* − *F* − α_2_*P*_2_. Workers' dissatisfaction with the tough punishment of safety managers will produce *A*_1_ psychological compensation under the workers' Passive response Strategy, and psychological compensation *A*_2_ for the foreman, the benefit is −α_2_*P*_3_ + *A*_1_ − *A*_2_.

(5) When the safety manager selects Appeasing supervision, the foreman selects Tough and the worker selects Active participation. Safety managers need to pay management costs and possible safety rewards or penalties, and their benefits are −*C*_12_ + (1 − α_1_)*I* − α_1_*P*_1_. The foreman needs to pay the management cost *C*_2_ and the income is −*C*_2_ − α_1_*P*_2_. The worker's income is −*T* + *A*_2_ − α_1_*P*_3_.

(6) When the safety manager selects Appeasing supervision, the foreman selects Tough and the worker selects Passive response. At this time, the safety risk will increase to α_2_. Safety managers need to pay management costs and possible safety rewards or penalties, and their benefits are −*C*_12_ + (1 − α_2_)*I* − α_2_*P*_1_. The foreman needs to pay the management cost *C*_2_ and the income is −*C*_2_ − α_2_*P*_2_. The worker's income is −α_2_*P*_3_ − *A*_2_.

(7) When the safety manager selects Appeasing supervision, the foreman selects Lenient and the worker selects Active participation. Safety managers need to pay management costs and possible safety rewards or penalties, and their benefits are −*C*_12_ + (1 − α_1_)*I* − α_1_*P*_1_. The foreman needs to bear the psychological pressure from the safety manager, and the profit is −*K* − α_1_*P*_2_. The worker's income is −*T* − α_1_*P*_3_.

(8) When the safety manager selects Appeasing supervision, the foreman selects Lenient and the worker selects Passive response. At this time, the safety risk will increase to α_2_. Safety managers need to pay management costs and possible safety rewards or penalties, and their benefits are −*C*_12_ + (1 − α_2_)*I* − α_2_*P*_1_. The foreman's income is −*K* − α_2_*P*_2_. The worker's income is −α_2_*P*_3_.

The predicted returns of the three groups under various situations were computed using the description in Table 3 and the evolutionary game analysis method that replicates the dynamics.

**Table 3:** Payoff matrix.

	**Strict supervision** **(x)**		**Appeasing supervision** **(1 − *x*)**	
	**Tough** **(y)**	**Lenient** **(1 − *y*)**	**Tough** **(y)**	**Lenient** **(1 − *y*)**
Active participation (*z*)	−*C*_11_ + (1 − α_1_)*I* − α_1_*P*_1_	−*C*_11_ + (1 − α_1_)*I* − α_1_*P*_1_	−*C*_12_ + (1 − α_1_)*I* − α_1_*P*_1_	−*C*_12_ + (1 − α_1_)*I* − α_1_*P*_1_
	−*R* − α_1_*P*_2_	*R* − *F* − α_1_*P*_2_	−*C*_2_ − α_1_*P*_2_	−*K* − α_1_*P*_2_
	−*T* − *F* − α_1_*P*_3_	−*T*+*A*_2_ − α_1_*P*_3_	−*T*+*A*_2_ − α_1_*P*_3_	−*T* − α_1_*P*_3_
Passive response (1 − *z*)	−*C*_11_ + (1 − α_2_)*I* − α_2_*P*_1_	−*C*_11_ + (1 − α_2_)*I* − α_2_*P*_1_	−*C*_12_ + (1 − α_2_)*I* − α_2_*P*_1_	−*C*_12_ + (1 − α_2_)*I* − α_2_*P*_1_
	−*R* − α_2_*P*_2_	*R* − *F* − α_2_*P*_2_	−*C*_2_ − α_2_*P*_2_	−*K* − α_2_*P*_2_
	−α_2_*P*_3_ + *A*_1_ − *F*	−α_2_*P*_3_ + *A*_1_ − *A*_2_	−α_2_*P*_3_ − *A*_2_	−α_2_*P*_3_

The expected payoffs for the manager's choice of the “Strict supervision” or “Appeasing supervision” strategy set are *V*_*x*_ and *V*_1−*x*_, respectively, and the average expected payoff is *V*_1_, then we have:

(1) $$\begin{array}{r} \begin{array}{c} V_{x}=yz\left(-C_{11}+\left(1-\alpha_{1}\right)I-\alpha_{1}P_{1}\right)\\ \;+\left(1-y\right)z\left(-C_{11}+\left(1-\alpha_{1}\right)I-\alpha_{1}P_{1}\right)\\ \;+y\left(1-z\right)\left(-C_{11}+\left(1-\alpha_{2}\right)-\alpha_{2}P_{1}\right)\\ \;+\left(1-y\right)\left(1-z\right)\left(-C_{11}+\left(1-\alpha_{2}\right)-\alpha_{2}P_{1}\right) \end{array} \end{array}$$

(2) $$\begin{array}{r} \begin{array}{c} V_{1-x}=yz\left(-C_{12}+\left(1-\alpha_{1}\right)I-\alpha_{1}P_{1}\right)\\ \;+\left(1-y\right)z\left(-C_{12}+\left(1-\alpha_{1}\right)I-\alpha_{1}P_{1}\right)\\ \;+y\left(1-z\right)\left(-C_{12}+\left(1-\alpha_{2}\right)I-\alpha_{2}P_{1}\right)\\ \;+\left(1-y\right)\left(1-z\right)\left(-C_{12}+\left(1-\alpha_{2}\right)I-\alpha_{2}P_{1}\right) \end{array} \end{array}$$

(3) $$\begin{array}{r} V_{1}=xV_{x}+\left(1-x\right)V_{1-x} \end{array}$$

The replicated dynamic equation for the manager's behavioral strategy is then:

(4) $$\begin{array}{r} f\left(x\right)=dx/dt=x\left(1-x\right)\left(C_{12}-C_{11}\right) \end{array}$$

Similarly, the expected benefits of the foreman choosing the “Tough” or “Lenient” strategy set are *V*_*y*_ and *V*_1−*y*_, respectively, and the average expected benefit is *V*_2_, then we have:

(5) $$\begin{array}{r} \begin{array}{c} V_{y}=xz\left(-R-\alpha_{1}P_{2}\right)+\left(1-x\right)z\left(-C_{2}-\alpha_{1}P_{2}\right)\\ \;+x\left(1-z\right)\left(-R-\alpha_{2}P_{2}\right)\\ \;+\left(1-x\right)\left(1-z\right)\left(-C_{2}-\alpha_{2}P_{2}\right) \end{array} \end{array}$$

(6) $$\begin{array}{r} \begin{array}{c} V_{1-y}=xz\left(R-F-\alpha_{1}P_{2}\right)\\ \;+\left(1-x\right)z\left(-K-\alpha_{1}P_{2}\right)\\ \;+x\left(1-z\right)\left(R-F-\alpha_{2}P_{2}\right)\\ \;+\left(1-x\right)\left(1-z\right)\left(-K-\alpha_{2}P_{2}\right) \end{array} \end{array}$$

(7) $$\begin{array}{r} V_{2}=yV_{y}+\left(1-y\right)V_{1-y} \end{array}$$

The replicated dynamic equation for the behavioral strategy of the foreman is then:

(8) $$\begin{array}{r} f\left(y\right)=dy/dt=y\left(1-y\right)\left(-C_{2}+K+\left(C_{2}+F-K-2R\right)x\right) \end{array}$$

The expected benefits of the workers choosing the “Active participation” or “Passive response” strategy set are *V*_*z*_ and *V*_1−*z*_, respectively, and the average expected benefit is *V*_3_, then we have:

(9) $$\begin{array}{r} \begin{array}{c} V_{z}=xy\left(-T-F-\alpha_{1}P_{3}\right)\\ \;+x\left(1-y\right)\left(-T+A_{2}-\alpha_{1}P_{3}\right)\\ \;+\left(1-x\right)y\left(-T+A_{2}-\alpha_{1}P_{3}\right)\\ \;+\left(1-x\right)\left(1-y\right)\left(-T-\alpha_{1}P_{3}\right) \end{array} \end{array}$$

(10) $$\begin{array}{r} \begin{array}{c} V_{1-z}=xy\left(-\alpha_{2}P_{3}+A_{1}-F\right)\\ \;+x\left(1-y\right)\left(-\alpha_{2}P_{3}+A_{1}-A_{2}\right)\\ \;+\left(1-x\right)y\left(-\alpha_{2}P_{3}-A_{2}\right)\\ \;+\left(1-x\right)\left(1-y\right)\left(-\alpha_{2}P_{3}\right) \end{array} \end{array}$$

(11) $$\begin{array}{r} V_{3}=zV_{z}+\left(1-z\right)V_{1-z} \end{array}$$

The replication dynamic equation for the worker's behavioral strategy is then:

(12) $$\begin{array}{r} \begin{array}{c} f\left(z\right)=dz/dt=z\left(V_{z}-V_{3}\right)\\ \;=z\left(1-z\right)\left(-\alpha_{1}P_{3}+\alpha_{2}P_{3}-T-A_{1}x+2A_{2}x+2A_{2}y-4A_{2}xy\right) \end{array} \end{array}$$

### 3.4. Equilibrium Analysis

Make the replication dynamic equations to 0, that is Equations 4, 8, 12 to 0. By solving it, we can get eight equilibrium points: *S*_1_(0, 0, 0), *S*_2_(0, 0, 1), *S*_3_(0, 1, 0), *S*_4_(1, 0, 0), *S*_5_(1, 1, 0), *S*_6_(1, 0, 1), *S*_7_(0, 1, 1), *S*_8_(1, 1, 1). From Friedman (1991), it is known that the stability of the replica dynamic equation is determined by the eigenvalue of the Jacobi matrix *J*. The system is asymptotically stable at the equilibrium point if all eigenvalues of the Jacobi matrix are negative real numbers, and unstable at the equilibrium point if the Jacobi matrix has at least one positive real number of eigenvalues. If the eigenvalues of the Jacobi matrix have imaginary roots while the other eigenvalues are real roots, the stability of the system cannot be judged at the equilibrium point. The Jacobi matrix is:

(13) $$\begin{array}{r} J=\left(\begin{array}{ccc} J_{11} & J_{12} & J_{13}\\ J_{21} & J_{22} & J_{23}\\ J_{31} & J_{32} & J_{33} \end{array}\right) \end{array}$$

(14) $$\begin{array}{r} J_{11}=\frac{\partial f\left(x\right)}{\partial x}=\left(C_{11}-C_{12}\right)\left(-1+x\right)+\left(C_{11}-C_{12}\right)x \end{array}$$

(15) $$\begin{array}{r} J_{12}=\frac{\partial f\left(x\right)}{\partial y}=0 \end{array}$$

(16) $$\begin{array}{r} J_{13}=\frac{\partial f\left(x\right)}{\partial z}=0 \end{array}$$

(17) $$\begin{array}{r} J_{21}=\frac{\partial f\left(y\right)}{\partial x}=\left(-C_{2}-F+K+2R\right)\left(-1+y\right)y \end{array}$$

(18) $$\begin{array}{r} J_{22}=\frac{\partial f\left(y\right)}{\partial y}=\left(C_{2}-K-\left(C_{2}+F-K-2R\right)x\right)\left(-1+2y\right) \end{array}$$

(19) $$\begin{array}{r} J_{23}=\frac{\partial f\left(y\right)}{\partial z}=0 \end{array}$$

(20) $$\begin{array}{r} J_{31}=\frac{\partial f\left(z\right)}{\partial x}=\left(A_{1}-2A_{2}+4A_{2}y\right)\left(-1+z\right)z \end{array}$$

(21) $$\begin{array}{r} J_{32}=\frac{\partial f\left(z\right)}{\partial y}=\left(-2A_{2}+4A_{2}x\right)\left(-1+z\right)z \end{array}$$

(22) $$\begin{array}{r} \begin{array}{c} J_{33}=\frac{\partial f\left(z\right)}{\partial z}\\ \;=\left(\alpha_{1}P_{3}-\alpha_{2}P_{3}+T+A_{1}x-2A_{2}x-2A_{2}y+4A_{2}xy\right)\left(-1+2z\right) \end{array} \end{array}$$

When the equilibrium point is brought into the Jacobian matrix, it can be found that except *J*_11_, *J*_22_, and *J*_33_ are all 0, that is, the Jacobian matrix of *S*_1_-*S*_8_ equilibrium point is diagonal matrix. That is, the values of *J*_11_, *J*_22_, and *J*_33_ are the eigenvalues of Jacobian matrix. On this basis, eight equilibrium points are brought into the Jacobian matrix to obtain the conditions required for equilibrium. As shown in Table 4.

**Table 4:** Equilibrium points stability analysis.

**Equilibrium points**	**λ_1_**	**λ_2_**	**λ_3_**
*S*_1_(0, 0, 0)	*C*_12_ < *C*_11_	*K* < *C*_2_	α_2_*P*_3_ < α_1_*P*_3_ + *T*
*S*_2_(0, 0, 1)	*C*_12_ < *C*_11_	*K* < *C*_2_	α_2_*P*_3_ > α_1_*P*_3_ + *T*
*S*_3_(0, 1, 0)	*C*_12_ < *C*_11_	*K* > *C*_2_	2*A*_2_ + α_2_*P*_3_ < *T* + α_1_*P*_3_
*S*_4_(1, 0, 0)	*C*_12_ > *C*_11_	*F* < 2*R*	2*A*_2_ + α_2_*P*_3_ < *T* + α_1_*P*_3_ + *A*_1_
*S*_5_(1, 1, 0)	*C*_12_ > *C*_11_	*F* > 2*R*	*a*_2_*P*_3_ < *A*_1_ + *a*_1_*P*_3_ + *T*
*S*_6_(1, 0, 1)	*C*_12_ > *C*_11_	*F* < 2*R*	*A*_1_ + α_1_*P*_3_ + *T* < α_2_*P*_3_ + 2*A*_2_
*S*_7_(0, 1, 1)	*C*_12_ < *C*_11_	*K* > *C*_2_	α_1_*P*_3_ + *T* < α_2_*P*_3_ + 2*A*_2_
*S*_8_(1, 1, 1)	*C*_12_ > *C*_11_	*F* > 2*R*	*A*_1_ + α_1_*P*_3_ + *T* < α_2_*P*_3_

(1) Whether, safety managers choose Strict supervision or Appeasing supervision because the rewards and punishments incurred due to the strengths and weaknesses of the safety climate are uncertain, what safety managers can determine is their perception of their own inputs. When *C*_11_ > *C*_12_, i.e., the safety managers choose the Strict supervision strategy more than the choice of the Appeasing supervision strategy.

(2) For the foreman, the key influential factors affecting his strategy choice were *K*, *C*_2_, *R*, and *F*. The strategy choice of the foreman is more dependent on the handling attitude of the safety managers. When safety managers choose a strict preservation strategy, the foreman weighs the penalty incurred under the managers against the magnitude of prestige within the working group. If the foreman pays more attention to the gains and losses of reputation, then tends to choose the lenient strategy, and vice versa tends to choose tough. When safety managers choose an opposing preservation strategy, the foreman ager weighs the amount of psychological stress imparted by the managers against the additional management paying management costs. If it is difficult for the foreman to assume the pressure of the safety managers, there is a greater tendency to opt for the lenient strategy and vice versa for the tough.

(3) Workers are full participants in the construction process and have more critical influencing factors in their strategy choice, α_1_, α_2_, *P*_3_, *T*, *A*_1_, *A*_2_. Once a safety accident occurs, it is a devastating disaster for workers, and the significance of life is paramount. Negative participation of workers in safety construction elevates the probability of safety incidents. In fact, construction engineering makes very much effort to construction safety, and workers' perception of safety incidents is weak. In this case, workers will have a greater perceived gravity of elevating additional safety construction costs. Workers' ethical approval is another large factor that significantly affects workers' behavior, which is specifically expressed in this paper as dissatisfaction *A*_1_ with managers, and psychological compensation *A*_2_ for the foreman. The analysis of influencing factors on worker's strategy selection is more complicated and will be further analyzed in the next section.

In summary, the safety managers' and foremen's perception of loss following an accident is not strong, largely because both parties are less involved in the construction process and are less sensitive to the importance of construction safety. The importance of a safe climate is strongly perceived by workers as direct participants. Excellent safety performance requires a concerted effort from stakeholders, and the most desirable combination of strategies is *S*_7_(0, 1, 1) and *S*_8_(1, 1, 1). But it is very difficult for all the participating founders to maintain a high level of safety awareness, the interests of the three parties are staggered, and when safety accidents do not occur, they pay more attention to their gains of interest. Many basic parameters cause changes in the strategies of the parties, and it is necessary to make an approximate prediction of the changes in the strategies caused by the parameters before the numerical simulation. The effects of changes in each parameter on the choice of the tripartite strategy are shown in Table 5 (effect on the values of *x*, *y*, *z*).

**Table 5:** The effects of changes in parameter.

**Parameter**	**Workers** **(z)**	**Foremen** **(y)**	**Safety managers** **(x)**
*C*_11_ ↑	-	-	→ 0
*C*_12_ ↑	-	-	→ 1
*F ↑ C*_2_ ↑	→ 0	→ 0	-
*K ↑ R ↑*	→ 1	→ 1	-
α_1_ ↑ α_2_ ↑	→ 1	→ 1	→ 1
*P*_3_ ↑ *T ↑ A*_2_ ↑	→ 1	-	-
*A*_1_ ↑	→ 0	-	→ 0

## 4. Simulation Analysis

### 4.1. Initialization Setting

Foremen and workers use active measures not only to ensure their own safety, but also to preserve their own psychological state of confrontation in the three parties, whereas managers are primarily concerned with punishment for negative effects. There are two main ways to motivate the team to choose a proactive strategy: first, through managerial pressure to keep the team at least above the safety baseline; and second, by playing a compensatory role (Schaumberg and Flynn, 2021) and raising their awareness to participate proactively. In Table 5, it can be seen that the game perceptions of the three groups can be adjusted by the parameters. Since this paper mainly discusses the state and influence of workers, we test how the key parameters affect the ESS of the previously proposed tripartite game model through simulation, and find the evolutionary path by adjusting the constraints, so as to promote the collective behavior to achieve the expected evolutionary stability and obtain excellent safety performance.

The reasonable simulation of three-party data is an important and complex problem, this paper selects a specific project located in Nanchang, China, a research center building. The main building of the research center was designed to have 24 floors and the podium was designed to have 2 floors, with only one tower crane used in each during construction. When the construction of the main building reached 6 floors, the construction of the podium started. As the working surface of the main building is small and adjacent to the podium, the construction teams are prone to conflicts over the use of materials or apparatus when the main building and podium are under construction at the same time, causing safety hazards. It is assumed that during the pouring of the top floor of the podium building, there was a conflict between the reinforcing steel team and the carpentry team due to the right to use the tower crane and the placement of the plates, and that the incident did not result in consequences such as injuries or damage to objects. The value of psychological factors is difficult to define, and this is the key of this paper. Therefore, in the numerical assignment of psychological factors, we choose to carry out long-span and multi-level simulation to ensure generality.

For clearer expression, we limit the setting of parameters to [0, 10]. These values only represent the direct relative relationship of parameters and do not represent practical significance. According to the above description, we consider *P*_3_ to be a large value, so we set *P*_3_ = 7. Workers' negative responses would make the incidence of safety accidents higher, so α_2_ > α_1_. In order to test the hypothesis and model analysis of evolutionary games, many researchers have used numerical simulation approaches for their studies. Based on the assumptions and analysis of the model in this paper, and in order to facilitate the subsequent numerical simulation of the parameters, we set the initialization of each parameter. The parameters are initially set as follows: *C*_11_ = 4, *C*_12_ = 4, *I* = 2, *F* = 3, *R* = 1.5, *K* = 3, *C*_2_ = 3.5, *P*_1_ = 2, *P*_2_ = 2, *T* = 2.8, α_1_ = 0.07, α_2_ = 0.35, *A*_1_ = 1, *A*_2_ = 1. In order to facilitate the subsequent numerical simulation calculation, the initialized parameter values are set relatively balanced, and to avoid the influence of subjective factors on the simulation results, we set the initial probability of the tripartite to 0.5.

In order to obtain a more realistic combination of strategies to choose from, the key influencing parameters will be adjusted. The impact of different parameter combinations on evolutionary stability is analyzed by simulating the following scenarios, and the constraints for maintaining effective security are explored.

### 4.2. The Influence of *C*_11_, *C*_12_, and *F*

**Figure 3:**
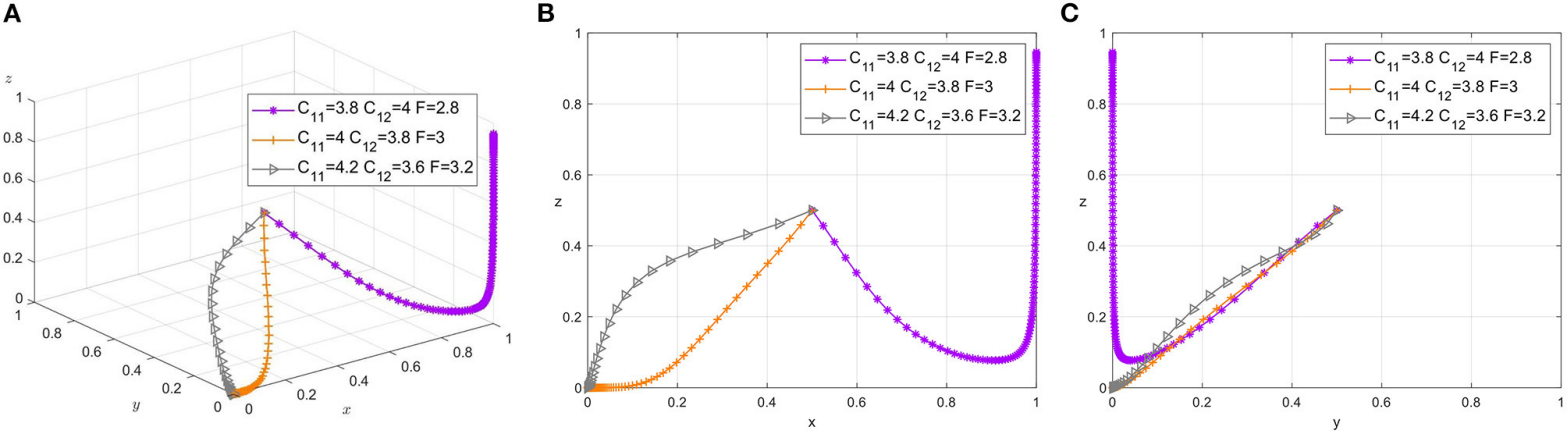
**(A)** Tripartite strategy evolution. **(B)** Safety managers-workers. **(C)** Foremen-workers.

The strategy selection of safety managers mainly depends on their own perception of management costs under the two strategies (*C*_11_, *C*_12_). Therefore, in this section, we choose to analyze the influence of changes in *C*_11_, *C*_12_, and *F* on three-party strategy selection. Parameter adjustment group, respectively: {*C*_11_ = 3.8, *C*_12_ = 4, *F* = 2.8}, {*C*_11_ = 4, *C*_12_ = 3.8, *F* = 3}, {*C*_11_ = 4.2, *C*_12_ = 3.6, *F* = 3.2}. Set The Times of simulation to 50 and the initial probability of all three parties to 0.5. The simulation results are shown in the Figure 3.

It can be seen that safety managers tend to choose Strict supervision strategy more when they choose Appeasing supervision strategy to pay more than Strict supervision. In this case, as the tough penalty measure *F* declines, workers are more likely to choose to approach safety construction aggressively, as shown in Figure 3B as well as Figure 3C. And in all three pre-determined situations, the foreman invariably chose to treat workers leniently. The decline in punishment makes the foreman take the punishment instead of the worker in order to maintain his own prestige, and the worker makes a positive response under the influence of the foreman. When safety managers are more inclined to choose Appeasing supervision strategy, foremen do not perceive punishment strongly, and foremen's perception of psychological pressure and management cost paid is a key factor in determining their strategy choice, which deserves further analysis and research.

### 4.3. The Influence of *R*, *C*_2_, and *A*_2_

**Figure 4:**
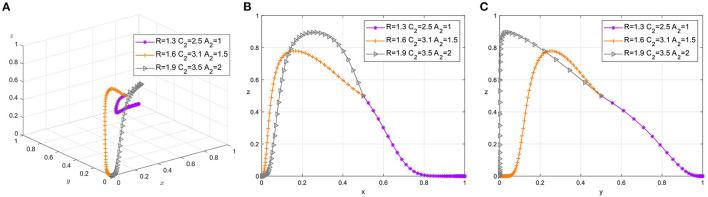
**(A)** Tripartite strategy evolution. **(B)** Safety managers-workers. **(C)** Foremen-workers.

Reputation gain or loss is an important influencing factor for foremen. The time-sensitive nature of construction projects dictates that the construction team and the project manager cannot always maintain a cooperative relationship and will part ways at the end of the construction project or even after the process is completed. However, the construction team will maintain lasting ties, and the foreman, as the top leader of the team, has an important prestige in the minds of the members. Therefore, in this subsection, we choose to analyze the impact of changes in *R*, *C*_2_, and *A*_2_ on the choice of tripartite strategies simultaneously. The parameter adjustment groups are: {*R* = 1.3, *C*_2_ = 2.5, *A*_2_ = 1},{*R* = 1.6, *C*_2_ = 3.1, *A*_2_ = 1.5},{*R* = 1.9, *C*_2_ = 3.5, *A*_2_ = 2}. In order to distinguish the difference between the two attitudes of the safety supervisor, set the first group *C*_11_ = 3.8, *C*_12_ = 4, and the last two groups are *C*_11_ = 4, *C*_12_ = 3.8. Set The Times of simulation to 50 and the initial probability of all three parties to 0.5. The simulation results are shown in the Figure 4.

When safety managers choose Appeasing supervision strategy and foremen perceive higher management costs of treating workers strictly, foremen tend to bear the pressure from safety managers alone and treat workers leniently. And with the increase of reputation gain or loss, the faster the rate of foreman tends to be tolerant treatment. At this time, as the workers' psychological compensation to the foreman increases, the workers will tend to choose to actively participate in the safety construction, but the strong punishment from the safety manager will still cause the workers' dissatisfaction, so the workers will turn to negative response after the short tendency to actively participate, as shown in Figures 4B,C.

### 4.4. The Influence of *K*, *T*, and *A*_1_

**Figure 5:**
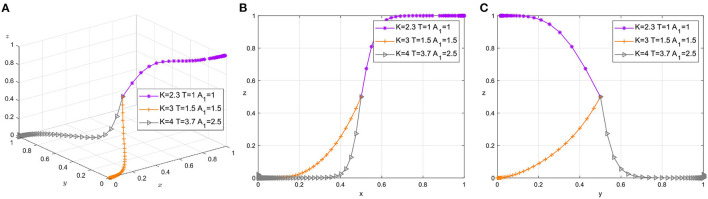
**(A)** Tripartite strategy evolution. **(B)** Safety managers-workers. **(C)** Foremen-workers.

Workers' behavior is influenced by their own psychological factors, especially the new generation of construction workers, whose psychological factors account for a higher percentage. Meanwhile, construction workers, as direct participants in the construction process, their attitudes toward the safety climate directly determine the safety performance. Therefore, in this subsection, we choose to analyze the effects of changes in *K*, *T*, and *A*_1_ on the choice of tripartite strategies simultaneously. The parameter adjustment groups were {*K* = 2.3, *T* = 1, *A*_1_ = 1}, {*K* = 3, *T* = 2.8, *A*_1_ = 1.5}, and {*K* = 4, *T* = 3.7, *A*_1_ = 2.5}. The number of simulations is set to 50, and the initial probabilities of all three parties are set to 0.5. The simulation results are shown in Figure 5.

As the foreman bears increased pressure from the safety manager, the foreman gradually tends to choose to treat the worker's management strictly, as shown in Figure 5A. With lower pressure from the safety manager, the foreman's choice still tends to be lenient, mainly due to the foreman's valuing of his position in the workgroup. The additional cost of workers' active participation in safety construction increases the tendency for workers to sit on their hands and dissatisfaction with safety managers induces workers to choose negative strategies, as shown in Figure 5B as well as Figure 5C.

In summary, we analyzed the stability of this evolutionary game model in the above three subsections in terms of the contrasting interests of safety managers, the profit and loss of foremen, and the psychological commitment of workers. In this paper, we set up two strategies for safety managers to manage conflict events, either Strict supervision strategy or Appeasing supervision strategy, foremen and workers need to respond to the way safety managers manage. To achieve the best safety performance, all three participants need to be positive about the safety climate. When the safety manager chooses the Strict supervision strategy and the foreman chooses himself or the worker to bear the punishment, the worker actively participates in the safety construction, i.e., S8(1,1,1), S6(1,0,1); when the safety manager chooses the Appeasing supervision strategy and the foreman actively conveys the management pressure to the worker, the worker actively participates in the safety construction, i.e., S7(0,1,1). However, when these strategy combinations occur, all three participants need to pay high management costs and additional costs, and the three participants will think that the occurrence of safety accidents is a very small probability out of luck, and consider more about their own benefit gain or loss and ignore the perception of project safety performance, as can be seen from the above simulation results, the occurrence probability of S6(1,0,1), S7(0,1,1), S8(1,1,1) strategy combinations do not occur with high probability. The analysis shows that the probability of foreman and worker choosing positive strategies is higher under the appeasing attitude of safety manager. The psychological factors of workers have a greater influence on their behavior, and safety managers can strengthen the safety management performance of the project by enhancing this aspect of management.

## 5. Discussion

(1) In this paper, we analyze the strategy choices of safety managers, foremen, and workers and their mutual influence through a three-way evolutionary game model. Based on the constructed payoff matrix, we obtained eight stable points: *S*_1_(0, 0, 0), *S*_2_(0, 0, 1), *S*_3_(0, 1, 0), *S*_4_(1, 0, 0), *S*_5_(1, 1, 0), *S*_6_(1, 0, 1), *S*_7_(0, 1, 1), *S*_8_(1, 1, 1). In the post-workgroup conflict safety management, we discarded the traditional choice of strict management strategy or regardless strategy for safety managers. We assume that safety managers must be involved in management, but two different management approaches exist, namely strict management and appeasing management. From the point of view of the safety of the whole project, the active participation of all three parties in safety management is the most idealized outcome. In this scenario, the safety manager will choose the strategy with the least perceived effort, and the active participation of foremen and workers in safety management will create a excellent safety climate, and the probability of safety accidents will be greatly reduced. However, this situation is too costly for all three parties and difficult to maintain. In the simulation results it is also found that the situations *S*_7_(0, 1, 1), *S*_8_(1, 1, 1) occur less frequently. However, the negative response of workers can lead to a large number of safety hazards, which is very detrimental to the safety performance of the project. In this paper, we mainly consider the influence of workers' psychological factors on their behavior, and promote the safety performance by influencing their psychological state. The following discussion focuses on the influence of the change of each parameter on the strategy choice of the three parties in order to achieve our expected strategy combination.

First, the rewards and penalties received by the safety manager are determined by the probability of a worker-induced safety incident, regardless of which management approach he or she chooses. The safety manager can determine only the management cost paid by each of his own choice of two strategies, *C*_11_, *C*_12_. The safety manager's strategy choice is mainly determined by these two parameters. However, the safety manager's management style largely influences the strategy choice of foremen and workers. For this reason the safety manager's payoff for both options can only be used as a reference to choose the management style that is more conducive to the active participation of foremen and workers.

Second, reputation is very important to the foreman. The team of workers and foremen together will continue to work for a long time, moving through multiple projects. In general, the leader's ability to control the team is very important, which can maintain a team's excellent performance (Zhu et al., 2021). This makes the foreman prefer to bear the punishment from the safety manager alone to be lenient with the workers within their own team. If the safety manager chooses a soft strategy and does not issue a strong monetary penalty to the work team, instead he applies psychological pressure on the foreman. Psychological pressure is to some extent a higher deterrent for the foreman because the construction project cycle is relatively long, and conflicts between the foreman and the safety manager can easily make the workgroup encounter various obstacles in the process. In this paper, different profit and loss measures of foremen under different attitudes of safety managers are established.

Furthermore, as the main builder of safety climate and the main participant of construction process, workers' behavior directly affects the probability of safety accidents. In order to explore the influence of workers' psychological factors on safety construction in an all-round way, we selected the indicators of dissatisfaction with safety managers and identification with foremen influenced by workers' moral identity, *A*_1_, *A*_2_. Through simulation we can find that the joint management of safety managers and foremen has a greater influence on workers. Workers' dissatisfaction with safety managers leads to their negative emotions, while the foreman's tolerant treatment leads to workers' psychological compensation and active participation in safety construction. At the same time, the direct victim of safety accidents is also the worker, and the perceived level of safety loss is also a significant influence on the workers' behavioral choices.

(2) In terms of safety in the construction industry, an excellent safety culture and safety climate can enable projects to achieve a high level of safety performance, and there is a constant confrontation between managers and construction teams in the construction of safety culture and climate (Al-Bayati, 2021). However, confrontation brings only hindrances to the operation of the project; it is the efficient collaboration of all parties involved that makes the project run smoothly. As a result, many academics have concentrated on how to mediate between project partners in order to improve the project's safety climate. Li et al. (2017) assessed the safety climate from three perspectives: workers, safety environment, and safety management, and proposed that the recognition of workers and the pressure given by managers have an impact on the safety climate indicators. Umar and Egbu (2018) further noted that managers' commitment, employees' empowerment, and workers' safety engagement can all have a major impact on the safety climate of construction projects. Personality requirements for construction workers have increased in the current era. Tough management tactics cause people to rebel, and emotionally-driven behaviors yield unfavorable outcomes, lowering managers' prestige and decreasing management effectiveness. In short, it is not advisable to use stereotypical management methods if managers want to achieve efficient management effectiveness as well as excellent safety performance, especially after a conflict incident, where the handling of the incident is as important as the emotional reassurance of construction workers.

(3) The construction industry is distinguished by a huge number of workers and a small number of supervisors. For incidents that do not result in serious consequences, managers are to some degree in a weaker position, they choose to avoid such events and thus reduce the potential for worker hostility or what other managers perceive as hidden losses. Even minor safety problems, have the potential to result in casualties. Owners and investors should invest more in safety, reduce managers' subjective awareness of risk ratings, and minimize ignorance of safety threats for a range of personal reasons in order to reduce safety risks.

To this end, we make the following suggestions: (1) Construction units should adopt more favorable rewards and more severe punishments according to the merits of safety managers' management performance in order to motivate safety managers to deal with safety hazards more seriously and responsibly. (2) Construction units should pay more attention to the importance of foremen in safety education and improve the role of foremen as intermediaries between safety managers and workers. (3) Construction units should pay attention to the impact of construction workers' mental health on safety behavior, and promptly deal with incidents that occur among workers to avoid very hidden and huge safety hazards caused by psychological problems.

## 6. Conclusion

In this paper, a tripartite evolutionary game model of post-conflict handling in a workgroup is developed to explore the effects of the level of moral identity of workers and the role of the leader of the workgroup, and the two attitudes of the manager (Strict supervision, Appeasing supervision) on the tripartite strategy, and providing some suggestions and references for finding the best way to handle the situation. During processing, workers' state adjustment is an important factor affecting project safety performance, and the use of evolutionary game theory allows for both targeted study of changes in important parameters, as well as intuitive exploration of the effects on tripartite behavior while parameters are changing.

The new generation of construction workers is self-aware and rebellious, and the attitude of managers toward workers affects their initial attitude and key parameters. Therefore, in the management measures, the past participation results and membership composition of the incoming team should be fully considered, instead of all tough penalties. In addition, it is important to improve the education of managers and construction teams, which is of great significance to maintain the safety performance of the project.

It is impractical to fully understand the behavioral orientation of managers and teams through a game. In this paper, we do not intend to analyze which strategy can achieve the best safety results through a game; instead, we introduce key variables under the highest safety performance strategy combinations and look for optimization measures to enhance management efficiency.

In the future, we will explore how more factors affect the safety performance and management efficiency of projects, especially the prevention of small incidents that may lead to serious accidents, such as conflicts between project hierarchical relationships. Future work will focus on using algorithms to analyze the cooperation and confrontation of project populations and to compare more accurately and clearly the differences between the proposed model and other models.

## Data Availability Statement

The original contributions presented in the study are included in the article/supplementary material, further inquiries can be directed to the corresponding author/s.

## Author Contributions

Both authors listed have made a substantial, direct, and intellectual contribution to the work and approved it for publication.

## Funding

This work was supported by Natural Science Foundation of Hunan Province of China (2015JJ2004), Natural Science Foundation of Hunan Province of China (2021JJ30746), and Science innovation project of Changsha University of Science and Technology (CXCLY2022011).

## Conflict of Interest

The authors declare that the research was conducted in the absence of any commercial or financial relationships that could be construed as a potential conflict of interest.

## Publisher's Note

All claims expressed in this article are solely those of the authors and do not necessarily represent those of their affiliated organizations, or those of the publisher, the editors and the reviewers. Any product that may be evaluated in this article, or claim that may be made by its manufacturer, is not guaranteed or endorsed by the publisher.
